# Progress in surgical management strategies after neoadjuvant therapy in breast cancer

**DOI:** 10.1080/07853890.2025.2559132

**Published:** 2025-09-14

**Authors:** Rongchen Zhu, Lingyu Kong, Shanping Sun

**Affiliations:** ^a^ Shandong Second Medical University, Weifang, Shandong Province, China; ^b^ Department of Breast Surgery, Linyi Cancer Hospital, Linyi, Shandong Province, China; ^c^ Department of Breast and Thyroid Surgery, Liaocheng People’s Hospital, Liaocheng, Shandong Province, China

**Keywords:** Breast neoplasms, neoadjuvant therapy, mastectomy, sentinel lymph node biopsy

## Abstract

**Objective**: To summarize recent advances in surgical management strategies for breast cancer following neoadjuvant therapy (NAT), focusing on the potential to de-escalate surgery and optimize timing and techniques.

**Methods**: This is a narrative review synthesizing current literature and clinical evidence on surgical approaches after NAT.

**Results**: Advances in NAT have increased pathological complete response (pCR) rates, creating opportunities to reassess surgical strategies. For exceptional responders, accurate non-surgical assessment of residual cancer could potentially allow for the omission of surgery. For other patients, optimal timing of surgery after NAT can improve outcomes. Surgical de-escalation is a key theme: breast-conserving surgery or oncoplastic techniques can avoid mastectomy for primary tumors, while sentinel lymph node biopsy and novel techniques like targeted axillary dissection can minimize the need for extensive axillary lymph node dissection.

**Conclusion**: The evolution of NAT necessitates a refined understanding of the necessity, timing, and extent of surgery. Future research should focus on improving patient selection criteria and refining surgical techniques to enhance patient outcomes by safely de-escalating surgical intervention.

## Key Messages

- Neoadjuvant therapy may be used for downstaging in some patients with breast cancer.
- The optimum timing of surgery after completion of neoadjuvant chemotherapy is 4–8 weeks.
- Breast conserving surgery after neoadjuvant therapy is safe and feasible in select patients.

## Introduction

Neoadjuvant therapy refers to preoperative systemic treatment for breast cancer. The National Comprehensive Cancer Network’s (NCCN) Clinical Practice Guidelines for breast cancer management recommend neoadjuvant therapy for patients with inoperable breast cancer or patients with operable breast cancer, including those with HER-2 positive or triple negative breast cancer staged at *T* ≥ 2 or *N* > 1, those with large primary tumors who wish to breast conserving, those with positive lymph nodes, or those requiring time to determine a surgical plan. Neoadjuvant therapy is not suitable for patients with extensive intraductal components, unclear extent of invasive carcinoma, and tumors that are not palpable or cannot be evaluated clinically [1].

Neoadjuvant therapy has evolved from administration of a single chemotherapy to administration of chemotherapy and HER-2 targeted therapy based on molecular subtypes of breast cancer. The indications for neoadjuvant therapy are expanding; currently, most of high-risk patients (staged at *T* ≥ 2 or *N* > 1) and patients with tumors > 2 cm in size with distinct biological features (HER-2 positive or triple negative breast cancer) receive neoadjuvant therapy. Changes in the diagnosis and management of breast cancer have led to disparate data on the necessity for surgery, the timing of surgery, the use of breast conserving surgery, and management of the axilla. Nevertheless, the ongoing discussions about the most suitable timing and scope of surgery emphasize the necessity for additional evidence to assist in making clinical decisions, especially considering the evolving molecular subtypes of breast cancer [2]. This narrative review was conducted to summarize progress in the field. The studies included in this review were chosen based on their relevance to current surgical practices after neoadjuvant therapy, emphasizing recent advancements and significant discoveries in the field [3].

### Surgical management after neoadjuvant therapy

Neoadjuvant therapy is associated with a high pathological complete response (pCR) rate (>50–70%), particularly for triple-negative or HER-2+ breast cancer. In exceptional responders to neoadjuvant therapy, surgery may be avoided if the presence of residual cancer can be accurately assessed using a nonsurgical approach, including minimally invasive image-guided needle biopsy or imaging. However, diagnostic tools may not accurately confirm and rule out residual disease in the breast after neoadjuvant therapy. In the RESPONDER trial, vacuum-assisted biopsy (VAB) did not reliably exclude residual disease (false negative rate [FNR]: 17.8%) in patients with breast cancer of all tumor biologic subtypes who received neoadjuvant therapy [4]. In a pooled analysis of data derived from patients with breast cancer treated with neoadjuvant chemotherapy at the Royal Marsden Hospital, Seoul National University Hospital and MD Anderson Cancer Center, image-guided biopsy had an FNR of 18.6% for detection of a residual imaging abnormality <2 cm [5]. In the MICRA (Minimally Invasive Complete Response Assessment) trial, breast pCR was not accurately identified by ultrasound-guided core biopsies (FNR 47%) in patients who achieved radiological partial (rPR) or complete response (rCR) on MRI after neoadjuvant therapy [6].

Further investigations are required to determine if minimally invasive techniques can be used to integrate a surgery-free treatment strategy for select patients with pCR after neoadjuvant therapy. Most recently, a machine learning algorithm (intelligent VAB) was shown to reliably exclude residual cancer after neoadjuvant therapy [2,7,8]. The machine learning algorithm was trained, tested, and validated using data from patients with cT1-3, cN0 or +, HER-2+, triple-negative, or high-proliferative luminal B-like breast cancer who underwent VAB prior to standard breast surgery. Intelligent VAB had an FNR of 0.0–5.2%, specificity of 37.5–40.0%, and area under the receiver operating characteristic curve of 0.91–0.92 for detecting residual cancer (ypT + or *in situ* or ypN+) after neoadjuvant therapy.

Eliminating breast and axillary surgery for exceptional responders to neoadjuvant therapy warrants further investigation in future trials. As the importance of local surgical treatment declines in breast cancer, percutaneous methods to treat local breast cancer, such as combined biopsy and imaging-guided microwave ablation, may become more widespread [8].

### The timing of surgical procedures relative to neoadjuvant therapy

Optimal planning of surgery for breast cancer relative to neoadjuvant therapy depends on disease presentation (e.g. size and molecular subtype) and patient preference (e.g. breast conserving surgery). In recent years, imaging biomarkers (the mammographic appearance of breast cancer, according to the site of tumor origin) have been increasingly used to guide treatment. Imaging biomarkers provide objective and quantitative measurements during the development of lesions, reduce the subjective bias of qualitative assessment, and provide insights into intratumoral heterogeneity.

The indication for and response to neoadjuvant therapy should be considered. Patient selection is essential for optimizing outcomes, and the clinical benefits, risks, and limitations of neoadjuvant therapy should be carefully evaluated. In some patients, neoadjuvant therapy may reduce tumor size and facilitate breast conserving surgery. In patients with luminal breast cancers, if neoadjuvant therapy is not effective, defined as ‘no significant clinical changes according to the Response Evaluation Criteria in Solid Tumors and World Health Organization criteria or pathological changes according to the Miller and Payne criteria’, definitive surgery may be considered after 2–3 cycles of chemotherapy. In patients with node negative, ER+, PR+, HER2-neu negative invasive breast cancer who intend to undergo breast conserving surgery by downstaging disease with neoadjuvant therapy, breast-conserving plastic surgery may be needed to obtain negative margins. Although clinical trials of neoadjuvant chemotherapy regimens have shown promising efficacy and survival data, more evidence is needed to highlight the advantages of neoadjuvant compared with adjuvant chemotherapy in patients with operable early breast cancer. In some cases, neoadjuvant chemotherapy may not improve prognosis, and breast-conserving surgery after tumor downstaging by neoadjuvant chemotherapy may increase the risk of local recurrence. Patients with chemotherapy-sensitive triple-negative breast cancer and HER-2 + breast cancer often relapse after neoadjuvant therapy and require surgery.

Evidence describing the associations between survival outcomes and the time since last dose of neoadjuvant chemotherapy to definitive surgery is scarce. A systematic review and meta-analysis showed that the optimum timing of surgery after completion of neoadjuvant chemotherapy is 4–8 weeks as it is associated with increased disease-free survival (DFS) and overall survival (OS). The meta- analysis included 5 studies. One study showed patients with breast cancer were unlikely to experience a clinically relevant change in residual tumor size with surgical intervals of 4–8 weeks [9]. A second study showed patients with Stage I-III breast cancer had similar recurrence-free survival (RFS) and OS with surgical intervals up to 8 weeks [10]. A third study showed patients with Stage II and III breast cancer, particularly those with ER+/HER-2+ breast cancers, had improved pCR with surgical intervals of 8 weeks. In all patients, DFS and OS were improved with surgical intervals of 4–7 weeks [11]. In a fourth study, women with a diagnosis of breast cancer experienced maximal benefit from the previous treatment with a surgical interval of 21 days, and time to surgery was an independent predictor of RFS and OS [12].

Given the increasing number of patients with breast cancer, it may become difficult to ensure that each patient is treated within an optimal time frame. Physicians must create treatment schedules that balance increasing clinical demands with the opportunity to achieve maximal patient benefit.

### Type of breast surgery

#### Breast surgery after neoadjuvant therapy

Current evidence for definitive surgery and axillary management strategies in breast cancer is based on large clinical trials; however, it may not be consistently applied in the clinical setting. The Early Breast Cancer Trial Collaborative Group (EBCTCG) meta-analysis of patient level data showed a higher frequency of breast conserving surgery in patients treated with neoadjuvant therapy compared to adjuvant chemotherapy (65% vs.49%) [13]. Neoadjuvant therapy can downstage large primary tumors to a volume that permits breast conserving surgery. Some studies suggest that local recurrence rates after neoadjuvant therapy and breast conserving surgery are high [2,14], but recent data show that the risk of local recurrence is mainly driven by tumor biology and is not associated with the choice of neoadjuvant or adjuvant therapy [3].

Patient selection for breast conserving surgery after neoadjuvant therapy should consider pre-treatment tumor characteristics such as lesion size, location, and molecular subtype, response to neoadjuvant therapy, and volume of residual disease. The surgical approach should also consider patient age and preference. Breast conserving therapy may be contraindicated in patients with large, poorly differentiated primary tumors, lobular carcinoma, large volume of residual disease, and non-concentric shrinkage during neoadjuvant therapy. Proper patient selection will ensure the neoadjuvant and breast conserving approach results in excellent outcomes.

Breast conserving surgery after neoadjuvant therapy may be considered when a tumor can be excised with clear margins. However, predicting and determining response to neoadjuvant therapy may be a challenge. Adjuvant radiotherapy may be necessary to eradicate microscopic residual tumor. Reconstructive procedures can improve patient quality of life, Oncoplastic breast surgery is used to conserve the breast while removing the cancer [15]. Oncoplastic breast surgery may lower positive margin and reoperation rates and have better postoperative patient satisfaction compared with traditional breast conserving surgery. The application of oncoplastic breast surgery after neoadjuvant therapy is increasing. Oncoplastic breast surgery requires appropriate pre-operative planning to understand the location and spread of the cancer, the use of volume displacement or volume replacement procedures, and careful follow-up to adequately screen for tumor recurrence.

#### Management of the axilla after neoadjuvant therapy

Neoadjuvant therapy impacts the status of the sentinel and axillary lymph nodes; however, this remains an important diagnostic measure and has a role in determining therapy, assuming the FNR is minimal. Sentinel lymph node biopsy (SNLB) is a satisfactory approach to obtaining axillary staging information in patients with clinically node-negative (cN0) disease and may have utility for staging of the axilla after neoadjuvant therapy. Strategies that minimize the likelihood of axillary lymph node dissection (ALND) decrease morbidity, such as lymphedema, in patients with breast cancer.

SLNB has utility for axillary staging of early breast cancer in patients with cN0 disease, but the feasibility and timing of SLNB in these patients are controversial. A meta-analysis showed that the SLN identification rate of SLNB after neoadjuvant chemotherapy was between 89% and 96%, and the FNR was between 13% and 17% [16], indicating that neoadjuvant chemotherapy had little effect on the identification of SLNs, but the FNR was unacceptable (FNR > 10%). The SENTINA, ACOSOG Z1071 and SN FNAC clinical trials also raised concerns over the FNR of SLNB in patients with cN0 disease who received neoadjuvant chemotherapy. A summary of these trials is provided in Table 1. The FNR for SLNB after neoadjuvant therapy may be caused by disruption of lymphatic structures, tissue fibrosis, and altered lymphatic reflux pathways [17–20].

**Table 1.** Trials of SLNB after neoadjuvant therapy that converted cN1 to cNO.

	Enrollment time	Research content	Axillary pCR rate	SLN detection rate	Overall false negative rate	Methods to reduce false negative rates	False negative rate
			41.00%	92.90%	12.6%(95%CI9.85–16.05)	Detection of more than 3 SLNs	9.10%
ACOSOG Z1071	2009–2011	False-negative rate of SLN after initial cN1 neoadjuvant surgery				Two methods of tracing	10.80%
*n* = 649						Resection of suspicious lymph nodes marked before treatment	6.80%
						IHC evaluation of ITCs and micrometastases in SLNs	8.70%
						Ultrasound evaluation of the axilla	9.80%
SENTINA	2009–2012	Detection rate and false negative rate of SLN before and after neoadjuvant surgery	52.30%	80.10%	14.2%(95%CI9.9**–**19.4%)	Detection of more than 3 SLNs	<10%
*n* = 592						Two methods of tracing	8.60%
SN FNAC	2009–2012	False**-**negative rate of SLN after neoadjuvant surgery for initial LN positivity	34.50%	87.60%	8.4%(95% CI2.4**–**14.4%)a	Detection of more than 3 SLNs	4.90%
*n* = 141						Two methods of tracing	5.20%

Various techniques have been used to improve SLN identification rates and reduce the FNR after neoadjuvant therapy in breast cancer. Traditional SNLB techniques use a single or combined procedure with blue dye and/or gamma probe localization. Newer tracers include indocyanine green, nanocarbon suspensions, superparamagnetic iron oxide, or ultrasound imaging with a microbubble contrast agent [21]. Targeted axillary dissection (TAD) combines SLNB with excision of biopsy-proven positive axillary nodes, which are marked (marked lymph node biopsy [MLNB]) before neoadjuvant therapy [22,23]. In a prospective registry study in patients with invasive breast cancer who underwent clip insertion into biopsy-confirmed positive lymph nodes, TAD was successful in 86.9% of patients and the SLN and MLN were identical in 64.8% of patients. The FNR was 7.2% for MLNB and 4.3% for TAD followed by ALND [24]. In another prospective study, the FNR of SLNB and ALND was 10.1% and the FNR of TAD and ALND was 2.0% in patients with nodal metastases confirmed on biopsy and a clip placed in the sampled node. Taken together, these data imply that if TAD shows no evidence of residual disease, complete ALND and its associated morbidity may be avoided; however, treatment may be escalated to surgery (ALND) or radiotherapy in patients who underwent TAD with residual disease in their specimens. Although the reviewed studies offer valuable insights into post-neoadjuvant surgical strategies, it is important to acknowledge several limitations. Numerous research suffers from the constraint of having small sample sizes, thus necessitating bigger, randomized controlled trials to substantiate these discoveries and enhance clinical standards [25]. This approach to escalating therapy should be considered even if residual disease is small, such as micrometastases or isolated tumor cells (ITCs) [26–30].

The ongoing AXSANA (AXillary Surgery After NeoAdjuvant Treatment) trial is investigating the optimal approach for surgical axillary staging in patients who convert from clinically positive to clinically negative lymph node status with neoadjuvant therapy (cN+ → ycN0) [31]. This prospective study is observational, and its primary endpoints include oncological outcomes. The Alliance A011202 clinical trial will target breast cancer patients with positive SLNB after neoadjuvant therapy and evaluate whether regional lymph node radiotherapy can replace ALND [32].

Not all patients with clinically node-positive breast cancer (cN1-2) treated with neoadjuvant therapy with cN + are suitable for post neoadjuvant SLNB. The sample size of patients with cN2 and above included in clinical trials such as ACOSOG-Z1071, SENTINA and FN SNAC is small and the evidence is insufficient, so ALND is recommended for patients with cN2 before neoadjuvant therapy.

### The value of multidisciplinary diagnosis and treatment mode

Clinicians should be aware of evidence describing the impact of neoadjuvant therapy on the surgical strategy for different molecular subtypes of breast cancer. The use of a multidisciplinary team (MDT) approach to the diagnosis and treatment of malignant tumors is endorsed in many countries. The emphasis is on collaborative decision-making between core team members of relevant specialties to make evidence-based recommendations for patient management. The characteristics of the individual patient and patient preferences should be considered when developing an individualized treatment plan that achieves maximal benefit, minimal side effects, and maintains patient quality of life. Most current research focuses on identifying novel molecular and genetic prognostic and predictive factors in breast cancer, but more studies investigating the impact of neoadjuvant therapy on surgery and outcomes in this patient population are warranted.

## Conclusions

Neoadjuvant therapy allows downstaging in some patients with breast cancer. Minimally invasive image-guided needle biopsy or imaging may be used to identify breast pCR and the necessity for surgery. The optimum timing of surgery after completion of neoadjuvant chemotherapy is 4–8 weeks as it is associated with increased DFS and OS. Breast conserving surgery after neoadjuvant therapy is safe and feasible in select patients. Oncoplastic breast surgery and breast reconstruction allow wide excision and satisfactory cosmetic results. Management of axilla after neoadjuvant therapy requires further investigation. Treatment options should be discussed using a multidisciplinary approach to ensure the delivery of safe, evidence-based, quality care. Nevertheless, the application of these sophisticated tactics in various clinical environments may encounter obstacles, such as limited resources and the requirement for specialized instruction. Tackling these obstacles will be essential in guaranteeing fair and equal access to the best possible breast cancer treatment.

## Data Availability

Data sharing is not applicable to this article as no new data were created or analyzed in this study.

## Abbreviations

pCR: Pathological complete response rate
VAB: Vacuum-assisted biopsy
FNR: False negative rate
rPR: Radiological partial
rCR: Complete response
DFS: Disease-free survival
OS: Overall survival
RFS: Recurrence-free survival
EBCTCG: Early Breast Cancer Trial Collaborative Group
SNLB: Sentinel lymph node biopsy
cN0: Clinically node-negative
ALND: Axillary lymph node dissection
TAD: Targeted axillary dissection
MLNB: Marked lymph node biopsy
ITCs: Isolated tumor cells
AXSANA: AXillary Surgery After NeoAdjuvant Treatment
